# Human Urine as a Noninvasive Source of Kidney Cells

**DOI:** 10.1155/2015/362562

**Published:** 2015-05-18

**Authors:** Fanny Oliveira Arcolino, Agnès Tort Piella, Elli Papadimitriou, Benedetta Bussolati, Daniel J. Antonie, Patricia Murray, Lamberthus van den Heuvel, Elena Levtchenko

**Affiliations:** ^1^Department of Development and Regeneration, Catholic University Leuven, Herestraat 49, 3000 Leuven, Belgium; ^2^Institute of Translational Medicine, University of Liverpool, Crown Street, Liverpool L69 3BX, UK; ^3^Center for Molecular Biotechnology, Department of Molecular Biotechnology and Health Sciences, University of Turin, Via Nizza 52, 10126 Turin, Italy

## Abstract

Urine represents an unlimited source of patient-specific kidney cells that can be harvested noninvasively. Urine derived podocytes and proximal tubule cells have been used to study disease mechanisms and to screen for novel drug therapies in a variety of human kidney disorders. The urinary kidney stem/progenitor cells and extracellular vesicles, instead, might be promising for therapeutic treatments of kidney injury. The greatest advantages of urine as a source of viable cells are the easy collection and less complicated ethical issues. However, extensive characterization and *in vivo* studies still have to be performed before the clinical use of urine-derived kidney progenitors.

## 1. Introduction

Currently, dialysis and kidney transplantation are the only successful therapies for patients suffering from chronic renal failure. Increasing shortage of donor organs for orthotopic kidney transplantation worldwide urges the need of alternative therapies. Using kidney progenitor cells might be an alternative approach of treatment in different kidney diseases [1]. Cells isolated from kidney tissue samples have the advantage of coming from a defined origin. However, they are only available in limited amounts and the life span of mature cells in culture is short, while repeated isolations of cells from the same donor are not allowed. On the other hand, kidney epithelia are exposed to continuous passage of filtrate, and thousands of living cells from healthy humans are excreted daily [2]. These exfoliated cells from urinary sediment can be isolated and cultured and include epithelial cells shed from different parts of the nephrons, ureters, bladder, and urethra [3] representing a limitless source of noninvasively harvested viable cells.

**Figure 1:**
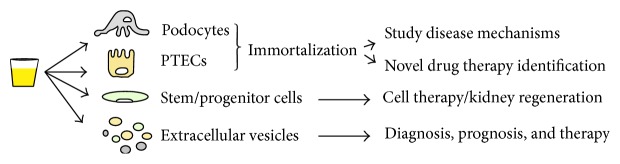
Urine as source of specific kidney cells and extracellular vesicles: applications and future perspectives.

The main types of exfoliated kidney cells in urine demonstrated so far are podocytes, proximal tubular cells, and undifferentiated cells called kidney stem/progenitor cells. Extracellular vesicles are also present in urine and can be an interesting source for studying the disease mechanisms and prognosis, as well as a potential regenerative stimulus through their paracrine effect [4] (Table 1 and Figure 1).

The use of urinary cells entails less ethical concerns and, most importantly, reduces immune response and rejection when applied in an autologous manner. As for all types of cells, prior to clinical use, further studies need to be performed to improve the isolation, culture, and differentiation steps to deliver cells with consistent number, quality, and stability.

## 2. Differentiated Kidney Cells Isolated from Urine

### 2.1. Podocytes

Podocytes are mature epithelial cells with a complex cellular organization consisting of a cell body, major processes, and foot processes. Interdigitating foot processes of neighboring podocytes form the slit diaphragm, and together they cover the outer part of the glomerular basement membrane playing a major role in establishing the selective permeability of the glomerular filtration barrier, which explains why podocyte injury is typically associated with marked proteinuria [5].

Since the 70s different techniques have been used for the isolation of podocytes directly from the glomeruli [6, 7]. However, podocytes can also be isolated from human urine, both from healthy subjects or patients with glomerulopathies, representing a noninvasive source of viable cells [8]. The quantification of podocytes in urine can be performed by immunofluorescence using specific antibodies such as anti-podocalyxin [9–11] or by isolation of the podocyte specific mRNA products (e.g., podocin or nephrin) [12].

Usually, higher numbers of podocytes are found in urine of patients with glomerular diseases compared to healthy subjects and those cells show faster* ex vivo* proliferation rate [11, 13]. For example, in patients with focal and segmental glomerulosclerosis (FSGS), podocyte loss increases in accordance to the level of injury and might be a marker of disease progression [11, 14]. Besides FSGS, urinary podocytes have been detected during the acute phase of other diseases, such as Henoch-Schönlein nephritis [10], IgA nephropathy [10], lupus nephritis [9, 15, 16], and also diabetic nephropathy [17] and preeclampsia [18]. Moreover, it has been suggested that urinary excretion of podocytes might be helpful to discriminate between acute and chronic stages of glomerular damage [19].

The loss of podocytes in urine has been also demonstrated in healthy individuals [14]. Interestingly, in healthy subjects most of the shed podocytes are senescent, while in experimental or human disease conditions a lot of viable podocytes are excreted [20].

Because of the limited proliferation rate and short life span of podocytes in culture, the immortalization step is instrumental for their maintenance. In 2002, Saleem et al. [21] developed a human conditionally immortalized podocyte cell line expressing specifically nephrin and podocin and later, in 2010, Sakairi et al. [22] created long-term urinary cell cultures from FSGS patients and healthy volunteers, showing that both cell lines present similar podocyte features [22]. These immortalized podocytes are transformed by insertion of a temperature-sensitive mutant of the proto-oncogene, SV40 large T antigen, so that they dedifferentiate and replicate under permissive conditions at 33°C, allowing unlimited turnover of cells, and regain a podocyte phenotype under nonpermissive conditions at 37°C [20].

### 2.2. Proximal Tubule Epithelial Cells (PTECs)

The proximal tubules are the primary targets in numerous inherited and acquired conditions such as in ischemic or toxic kidney injury or genetic Fanconi syndromes [23].

In 1991, Racusen et al. [24] were the first to show that viable PTECs could be isolated from human urine of patients with nephropathic cystinosis, a lysosomal storage disease causing renal Fanconi syndrome. Dörrenhaus et al. [3], in 2000, described proximal tubules urine cell colonies designated as type-2 colonies with a cobblestone-like morphology. These cells were able to form domes caused by the transepithelial fluid transport from the medium to the area between the culture plate surface and the cells monolayer [3]. Later on, three-dimensional collagen gel cultures were established using PTECs from human urine to drive them to a highly polarised state. And because the cells still had some proliferative potential and became polarized, they were able to form organised structures resembling the* in vivo* tubules [25].

Interestingly, the number of urinary PTECs does not always correspond to the degree of kidney injury, as for example, in primary hyperoxaluria; a higher number of exfoliated PTECs in urine were not detected [26]. Anyhow, lots of viable proximal tubule cells are voided in human urine and they can be exploited for physiological studies such as analysing the transport of drugs and different substances in the proximal tubules. Again, the limitations of using PTECs in culture are the limited cells number and short life span that can be overcome by the conditional immortalization of the cells. The first immortalized proximal tubule cell line was generated by Racusen et al. in 1995 [27]. The next cell line was only established in 2010, when Wilmer et al. [28] developed conditionally immortalized PTECs from urine of healthy subjects. These cells expressed multiple endogenous organic ion transporters, mimicking renal reabsorption and excretion. Shortly afterwards, another conditionally immortalized PTECs line was established from the urine of cystinotic patients [23].

Conditionally immortalized PTECs are useful models to explore the mechanisms involved in specific proximal tubular renal pathologies. Gorvin et al. [29] have used urinary PTECs lines of patients with Dent's disease to study receptor mediated endocytosis and endosomal acidification depending on the type of mutation in the* CLCN5* gene causing this condition. Urinary PTECs have been also applied for* in vitro* studies of cell physiology and toxicology, including the influx and efflux of drugs [28], and might represent a promising step towards a bioartificial kidney device [30]. In 2004, an FDA-approved phase I/II clinical trial that was performed in 10 patients using PTECs harvested from human kidneys in a bioartificial kidney demonstrated that the addition of human PTECs to replacement therapy improves metabolic activity with systemic effects in patients with acute renal failure and multiorgan failure [31]. Later, in 2008 a phase II randomized trial using the same device with nonautologous PTECs showed more rapid recovery of kidney function in critically ill patients with acute renal failure [32]. Presenting similar genetic and functional characteristics as kidney harvested PTECs [30], the urinary cells might have great advantage of possible autologous cell therapy that has to be further evaluated.

## 3. Undifferentiated Kidney Cells Isolated from Urine

During nephrogenesis, stem/progenitor cells are located in the cap mesenchyme and behave as true committed stem cells, capable of self-renewing and differentiation into different types of nephron epithelia [33, 34]. These cells express specific renal progenitor cells markers as SIX2 [34], Cited1 [33], NCAM, ep-CAM, and FZD7 [35] and have been extensively characterized. As amniotic fluid (AF) is mainly composed of fetal urine and lung exudates [36], it is believed that some subpopulations of AF cells are of kidney origin and are committed to renal fates. Indeed, amniotic fluid is believed to be an important source of stem cells in an intermediate stage between embryonic stem cells and adult stem cells. Da Sacco et al. [37] have successfully isolated a subpopulation of metanephric mesenchyme-like cells from AF and later showed that these cells are committed to nephron lineages, being capable to differentiate into functional podocytes [38]. These cells represent a new model to study podocyte cell biology and development. Additionally, the isolated renal committed stem cells from AF can be an attractive source of cells to repair kidney injury.

It is known that, in humans, nephrogenesis is completed at about 34–36 weeks of gestational age. Thus, the presence of stem/progenitor cells in the adult human kidney is highly discussed, as well as their possible origins. Initially, CD133+ cells presenting progenitor cells characteristics were isolated from adult renal cortex [39]. Subsequently, these cells were found in different segments of the nephrons as the urinary pole of the Bowman's capsule [40], in the proximal tubules and the inner medullary papilla region, including Henle's loop and the S3 limb segment [41]. In agreement with the idea of an existent stem cell-like population in mature kidneys, it has been demonstrated that freshly voided urine [42] and urine from the upper urinary tract [43] contain stem cells capable to reconstruct urological tissues. Contrasting these results, other groups have shown that the repair of acute injured renal tubules does not involve specialized kidney progenitors [44] but occurs from resident differentiated tubular cells that had survived the injury and underwent dedifferentiation [45] in response to damaging factors that may give them a higher proliferation capacity, the ability to redifferentiate and reintegrate the injured site. Regarding podocyte regeneration, it has been shown using linage fate tracing that cells of renin lineage from juxta-glomerular apparatus might represent progenitor cells in glomerular disease [46, 47].

The effectiveness of kidney stem/progenitor cell transplantation in animal models of kidney injury has been described using tissue progenitor cells from embryonic [48] and adult kidneys [47, 49]. Human embryonic nephron progenitor NCAM+ cells were engrafted and integrated in diseased murine kidneys and had beneficial effects on renal function halting disease progression in the 5/6-nephrectomy kidney injury model [48]. The injection of adult kidney CD133+CD24+PDX− cells in an adriamycin-induced nephropathy mouse model showed reduced proteinuria and improved chronic glomerular damage [47], while CD133+CD24+CD106+ cells injected in SCID mouse with acute tubular injury were able to generate novel tubular cells and improve renal function [49]. Moreover, the therapeutic effect of urine-derived stem cells was tested on athymic mouse model and VEGF-expressing urine-derived stem cells combined with human umbilical venous endothelial cells were used for treating vesicoureteral reflux and stress urinary incontinence [50]. These cells have also been effective in the development of a multilayer mucosal structure similar to that of native urinary tract tissue when seeded on 3D porous small intestinal submucosa scaffold and may serve as an alternative cell source in cell-based tissue engineering [51]. However, the therapeutic potential of urinary KSPCs in renal injury still has to be studied.

Altogether, these studies confirm the importance of urine as a noninvasive source of viable cells with potential for regenerative medicine and tissue engineering, in addition to cytotoxicity and pharmacological studies.

## 4. Urinary Extracellular Vesicles (EVs)

Extracellular vesicles (EVs) are small particles (100–1000 nm) secreted by all types of cells under both physiological and pathological conditions. They are composed by a lipid bilayer, which encloses several cytoplasmic proteins, lipids as well as nucleic acids, comprising their biological “cargo” [52–54]. Due to their apparently important role in cell-cell communication, EVs have gained an increasing interest during the last decades whereas numerous studies demonstrate their isolation from various body fluids, including urine [53]. As the content of EVs may reflect both the cell of origin and its pathophysiological state, urinary EVs represent a unique source of information for diagnostic purposes and may possibly display therapeutic functions along with stem/progenitor cells.

Recent studies provide evidence that uEVs present in the preurine may transfer information within the nephron segments, thus representing a mechanism of intranephron communication [55, 56]. Urinary EVs derive from every epithelial cell of the kidney, including renal progenitor cells. In this regard, Dimuccio et al. showed absence of CD133+ urinary EVs in patients suffering from end stage kidney disease, underlying the possible exhaustion of CD133+ progenitors in these patients [57]. Moreover, lower levels of CD133+ uEVs were present in delayed graft compared to early graft recovery, suggesting a possible correlation between levels of CD133+ vesicles in urine and the renal homeostasis or recovery after injury [57].

Finally, EVs are known to recapitulate the therapeutic effect of stem cells, due to their paracrine effects, resulting in a horizontal transfer of mRNA, microRNA, and proteins [52, 58–60]. In particular, mesenchymal stem cell-derived EVs stimulated proliferation and apoptosis resistance of tubular epithelial cells* in vitro* [59] and accelerated the morphological and functional recovery* in vivo* in different experimental animal models of renal injury [58, 60]. Within the urinary EVs, it is therefore possible that progenitor-derived EVs may be involved in local paracrine effect on neighboring cells, directing differentiation or regenerative programs.

Therefore, considering that every epithelial cell of the kidney may secret vesicles into the urinary space, urinary EVs may be used as markers of prognosis, diagnosis, and therapy of several kidney diseases.

## 5. Advantages, Limitations, and Future Challenges

The use of urine as source of kidney cells has great advantages compared to tissue harvesting due to the noninvasive methods of collection; it raises less ethical concerns, once urine is a excreted product of the body; many samples can be collected from the same individual, allowing investigation of disease progression and its treatments; cells could be used for autologous therapy avoiding immune rejection after transplantation due to antigenic differences; and very importantly, the cells collected in urine are viable, are able to proliferate in culture, and present similar features of cells harvested from kidney tissue.

However, culture of kidney cells presents some limitations because of the maturity of the cells. If urinary kidney stem/progenitor cells were able to differentiate into fully mature and functional kidney cells, they could overcome this problem.

In the future though, human renal cells isolated from urine might play a role in tissue engineering for personalized medicine in patients suffering from nephropathies or chronic renal disease. Bioartificial kidneys in combination with autologous kidney cells could help to improve kidney function and its outcomes. However before that, a full characterization and a rigorous selection of the cells should be done for safe implementation in clinical applications.

## Figures and Tables

**Table 1:** Types of kidney cells exfoliated in urine and their current applications.

Urine-derived kidney cell	Markers of disease activity	Disease modeling	Studying cell biology/physiology	Therapeutic effects
Podocytes	(i) Diabetic nephropathy [13, 17] (ii) Membranous nephropathy [61] (iii) Focal and segmental glomerulosclerosis [11] (iv) Henoch-Schönlein nephritis (v) IgA nephritis (vi) Lupus nephritis [9, 15] (vii) Preeclampsia [18] (viii) D + HUS [62] (ix) Diffuse mesangial sclerosis [63]	(i) Lupus nephritis [16]	(i) Characterization [22] (ii) Function [64, 65]	Not studied

PTECs	(i) Acute tubular necrosis [66, 67] (ii) Diabetes mellitus [67]	(i) Cystinosis [23, 24, 27] (ii) Diabetes mellitus [67] (iii) Hyperoxaluria [26] (iv) Dent disease [29] (v) Lowe syndrome [68]	(i) Characterization [30] (ii) Function [28, 30, 69]	(i) Paracrine effects of conditioned medium [70]

Stem/progenitors	Not studied	Not studied	(i) Characterization [37, 38, 42, 43, 71, 72]	(i) Differentiation into glomerular cells [38, 73] (ii) Genitourinary tissue reconstruction [74–76] (iii) Skeletal muscle regeneration [50, 77, 78] (iv) Neurologic tissue reconstruction [79]

Extracellular vesicles	(i) Focal and segmental glomerulosclerosis [80, 81]	Not studied	(i) Characterization [53, 81–84] (ii) Function [53]	(i) Kidney transplantation [57]
